# Allele-specific silencing of mutant p53 attenuates dominant-negative and gain-of-function activities

**DOI:** 10.18632/oncotarget.6634

**Published:** 2015-12-16

**Authors:** Swathi V. Iyer, Alejandro Parrales, Priya Begani, Akshay Narkar, Amit S. Adhikari, Luis A. Martinez, Tomoo Iwakuma

**Affiliations:** ^1^ Department of Cancer Biology, The University of Kansas Cancer Center, University of Kansas Medical Center, Kansas City, KS, USA; ^2^ Center for Advanced Preclinical Research, Center for Cancer Research, National Cancer Institute, Frederick, MD, USA; ^3^ Department of Pathology, Stony Brook School of Medicine, Stony Brook, NY, USA

**Keywords:** mutant p53, allele-specific siRNA silencing, gain of function, oncogene addiction, dominant negative

## Abstract

Many p53 hotspot mutants not only lose the transcriptional activity, but also show dominant-negative (DN) and oncogenic gain-of-function (GOF) activities. Increasing evidence indicates that knockdown of mutant p53 (mutp53) in cancer cells reduces their aggressive properties, suggesting that survival and proliferation of cancer cells are, at least partially, dependent on the presence of mutp53. However, these *p53* siRNAs can downregulate both wild-type p53 (wtp53) and mutp53, which limits their therapeutic applications. In order to specifically deplete mutp53, we have developed allele-specific siRNAs against p53 hotspot mutants and validated their biological effects in the absence or presence of wtp53. First, the *mutp53*-specific siRNAs selectively reduced protein levels of matched p53 mutants with minimal reduction in wtp53 levels. Second, downregulation of mutp53 in cancer cells expressing a mutp53 alone (p53^mut^) resulted in significantly decreased cell proliferation and migration. Third, transfection of *mutp53*-specific siRNAs in cancer cells expressing both wtp53 and mutp53 also reduced cell proliferation and migration with increased transcripts of p53 downstream target genes, which became further profound when cells were treated with an MDM2 inhibitor Nutlin-3a or a chemotherapeutic agent doxorubicin. These results indicate that depletion of mutp53 by its specific siRNA restored endogenous wtp53 activity in cells expressing both wtp53 and mutp53. This is the first study demonstrating biological effects and therapeutic potential of allele-specific silencing of mutp53 by *mutp53*-specific siRNAs in cancer cells expressing both wtp53 and mutp53, thus providing a novel strategy towards targeted cancer therapies.

## INTRODUCTION

Targeted cancer therapies are treatments that aim at specific characteristics of cancer cells, including proteins or pathways that provide cancer cells with surviving and proliferation signals [1]. Oncogenes play major roles in providing such signals, and hence depletion of oncogenes in cancer cells leads to attenuation of proliferation, survival, and tumor progression, suggesting that progression of cancer cells is frequently dependent on the presence of oncogenes [2]. Therapeutic strategies targeting oncogenes and their associated pathways would significantly increase specificity of treatments and improve efficacy of cancer therapy with reduced side-effects [1].

Approximately 50% of human cancers have mutations in the tumor suppressor *p53* gene, the majority of which are missense mutations [3, 4]. These p53 mutants frequently show oncogenic gain-of-function (GOF) activities, such as enhanced metastatic potential and drug resistance [5-8]. Our previous findings indicate that accumulation of GOF mutant p53 (mutp53) in cells is crucial for employing its oncogenic activity [9]. Importantly, knockdown of p53 mutants by *p53* siRNAs or shRNAs attenuates proliferation, drug resistance, and tumor development of cancer cells carrying mutp53 alone (p53^mut^), suggesting that survival and proliferation of cancer cells are dependent on the presence of GOF p53 mutants [7, 8, 10-14]. However, these *p53* siRNAs or shRNAs are not specific for mutp53 and can knockdown both wild-type p53 (wtp53) and mutp53. Thus, it is important to develop strategies that specifically deplete mutp53 for cancer therapy.

The siRNA technology offers an efficient and convenient strategy to deplete proteins of interest. The extraordinary sequence specificity of siRNA makes it an attractive tool for targeted cancer therapies. There are several reports demonstrating effectiveness of allele-specific siRNA oligonucleotides to specifically deplete mutant proteins that include EGFR V843I [15], keratin 6a N171K [16], TGFBI R124C [17], Tau V337M [18], and K-RAS G12V [19]. Most relevantly, Martinez *et al.* [20] designed a siRNA specific to p53^R248W^. They demonstrated that p53^R248W^ knockdown by *p53^R248W^*-specific siRNA induced apoptosis in MDAH087 cells carrying only p53^R248W^ and increased p21 protein levels and MDM2 promoter activity in p53-null H1299 cells transfected with both wtp53 and mutp53 [20]. However, no biological consequence of mutp53-specific knockdown in genuine heterozygous cells endogenously expressing both wtp53 and mutp53 was shown [20].

Our hypothesis states that specific downregulation of oncogenic mutp53 in cancer cells reduces malignant characteristics of cancer cells. To test this hypothesis and extrapolate the idea of mutp53 silencing for cancer therapy, we developed novel siRNAs specific for two hotspot p53 mutants, p53^R175H^ and p53^R273H^. Using these siRNAs, as well as *p53^R248W^*-specific siRNA, we examined the effects of allele-specific silencing of oncogenic p53 mutants on biological properties of cancer cells expressing mutp53 alone, as well as those expressing both wtp53 and mutp53. Our studies would be critical for developing novel strategies to specifically deplete mutp53 in cancer cells with little effect on wtp53, thereby having minimal side effects.

## RESULTS

### Downregulation of mutp53 reduced sphere- and tumor-forming potential of p53^mut^ cancer cells

**Figure 1:**
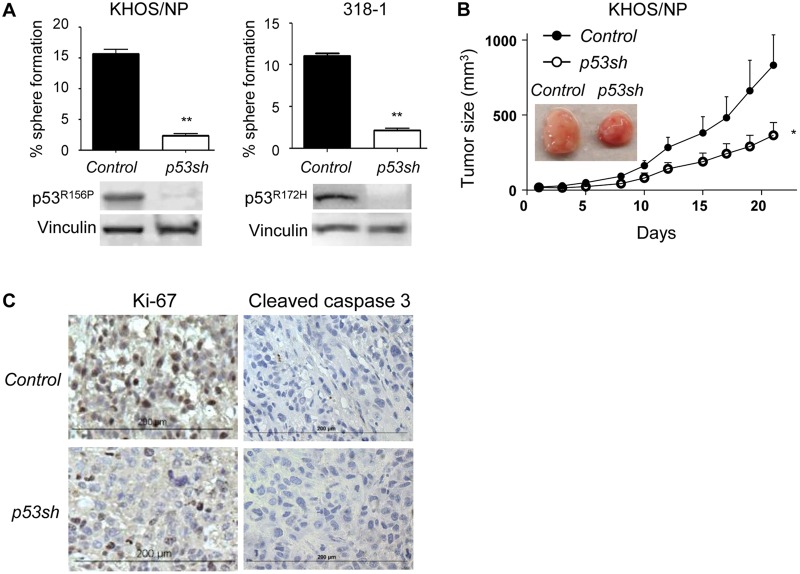
Mutp53 downregulation by *p53* shRNA inhibited malignant properties of cancer cells **A.** Sphere formation assays were performed using KHOS/NP (p53^R156P^) and 318-1 (p53^R172H^) cells infected with control empty or *p53* shRNA-encoding lentiviral vectors. Graph showing % of sphere formation (# of spheres formed/# of cells seeded) and representative western blotting for p53 and Vinculin is below the graphs. **B.** Control (*Control*, filled circle) or p53-downregulated (*p53sh*, open circle) KHOS/NP cells (1,000,000) were subcutaneously injected into NIH-III nude mice, and tumor sizes were measured three-dimensionally 3-4 times a week for 3 weeks (*n* = 6). Representative images of formed tumors are shown in the panel. Error bars: means ± S.D. *P < 0.05, **P < 0.01; Student's *t* test. **C.** Tumors formed in mice in Figure 1B were examined for the expression of Ki-67 and cleaved caspase-3 by immunohistochemistry.

Downregulation of mutp53 attenuates tumor progression due to loss of oncogenic GOF activity and/or addiction of cancer cells to mutp53 in several cancer types [10-14]. To confirm this, we examined effects of mutp53 knockdown by using *p53* shRNA-encoding lentiviral vectors which could downregulate both wtp53 and mutp53 on malignant properties of p53^mut^ cancer cells [21]. We first tested effects of mutp53 knockdown on the ability of cancer cells to grow in an anchorage- and serum-independent manner and form spheres, since cancer cells within spheres that could overcome anoikis (anchorage-dependent cell death) and proliferation arrest induced by loss of attachment and serum depletion are enriched within cells having high malignant properties, hence being well correlated with aggressive properties of cancer cells [22, 23]. Downregulation of p53^R156P^ in human KHOS/NP and p53^R172H^ in mouse 318-1 osteosarcoma cell lines significantly inhibited sphere formation (Figure 1A). Also, p53^R156P^ knockdown in KHOS/NP cells inhibited subcutaneous tumor growth in immunocompromised mice (Figure 1B). Immunohistochemistry of KHOS/NP-derived tumors revealed that p53^R156P^ knockdown resulted in reduced Ki-67 levels with little change in cleaved caspase-3 in tumors, suggesting reduction of tumor proliferation. These results suggest that progression of cancer cells is, at least partially, dependent on the presence of oncogenic mutp53.

### Identification of allele-specific siRNAs against p53^R273H^ and p53^R175H^

Although we found that mutp53 downregulation reduced malignant properties of cancer cells, the drawback of this strategy is that the *p53* shRNAs used could downregulate wtp53 along with mutp53. Hence, it is crucial to develop siRNAs that specifically knockdown mutp53 alone without affecting wtp53.

**Figure 2:**
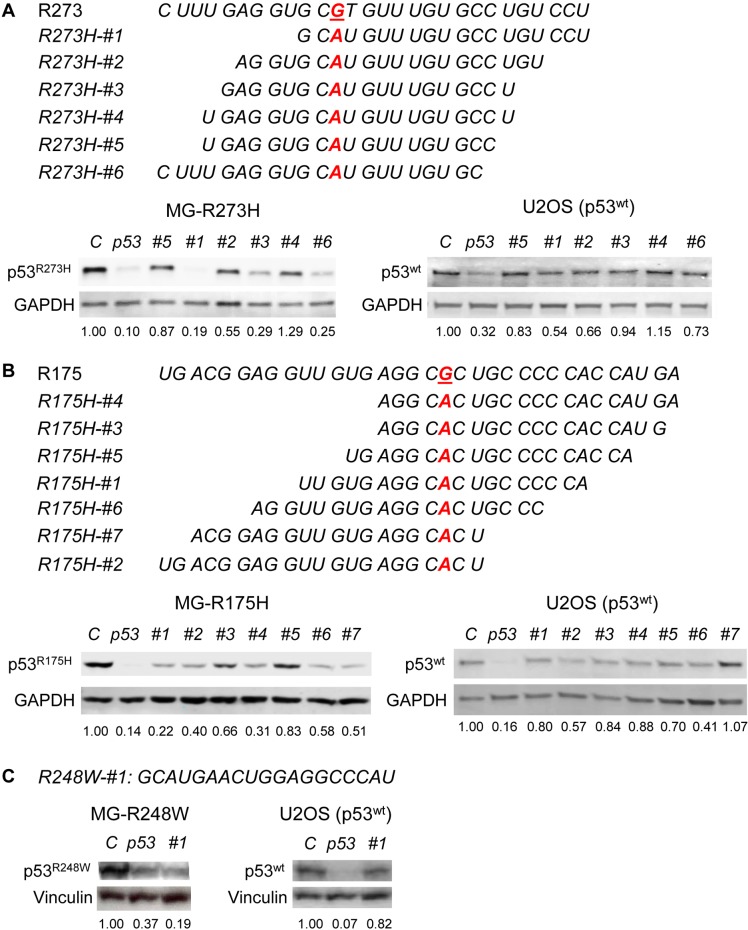
Design and identification of *mutp53*-specific siRNAs RNA sequences of human p53 surrounding codon R273 and target sequences for p53^R273H^ (*R273H-#1∼6*, A), codon R175 and target sequences for p53^R175H^ (*R175H-#1∼7*, B), and codon R248 and the target sequence for p53^R248W^ (*R248W-#1*, C). Representative western blotting results for p53 and GAPDH or Vinculin (loading controls) using MG-R273H **A.**, MG-R175H **B.**, and MG-R248W cell lines **C.**, as well as U2OS cell line, are shown below the target sequences. Relative p53 intensities to the control siRNA standardized by those of GAPDH or Vinculin are shown below the blots. C: *control* siRNA, *p53*: *p53* siRNA.

Mutations at codon 273 of p53 are one of the most frequent events in various types of human cancer (http://p53.fr/). Specifically, arginine (R) to histidine (H) missense mutant (p53^R273H^) is best characterized for its oncogenic GOF activity. We therefore attempted to identify a specific siRNA against p53^R273H^ having little effect on wtp53. We designed 6 different siRNAs against p53^R273H^ (Figure 2A). These siRNAs, as well as *non-target#1* siRNA (negative control, *C*) and a *p53* siRNA (positive control, *p53*), were transiently transfected into *p53*-null MG63 osteosarcoma cell line infected with a retroviral vector encoding *p53^R273H^* (MG-R273H, Supplementary Figure S1) or U2OS osteosarcoma cell line endogenously expressing wtp53, followed by western blotting for p53 (Figure 2A). Of these 6 siRNAs, *R273H-#1*, *3*, and *6* efficiently downregulated p53^R273H^, but *R273H-#3* had minimal effects on wtp53. Hence, we used *R273H-#3* for all the further experiments. Interestingly, *R273H-#2* and *4* have similar target sequences to that of *#3*, but they failed to efficiently knockdown p53^R273H^, suggesting precise and exquisite sequence specific nature of siRNA to downregulate expression of a target protein.

We next attempted to identify a siRNA specific to p53^R175H^, another hotspot p53 mutation with oncogenic GOF activity. We designed 7 different siRNAs against p53^R175H^ and transfected them into MG63 cells infected with a retroviral vector encoding *p53^R175H^* (MG-R175H, Supplementary Figure S1A) and U2OS. Western blotting results revealed that *R175H-#1, 2, 4, 6, and 7* efficiently downregulated p53^R175H^. Since densitometric analyses revealed that *R175H-#4* showed sufficient reduction in p53^R175H^ levels with minimal effects on the wtp53 level (Figure 2B), we used *R175H-#4* in all experiments to knockdown p53^R175H^ onward.

We also confirmed that transfection of the previously identified *p53^R248W^*-specific siRNA (*R248W-#1*: *GCAUGAACUGGAGGCCCAU*) resulted in successful downregulation of p53^R248W^ in MG-R248W cells (Supplementary Figure S1) without affecting wtp53 levels in U2OS cells (Figure 2C) [20].

To confirm that a mutant-specific siRNA could downregulate only the matched p53 mutant with little effect on other p53 mutants as in the case of wtp53, we transfected *non-target#1* (negative control), *R175H-#4* (positive control), *R273H-#3*, and *R248W-#1 siRNAs* into CAL33 cells endogenously expressing p53^R175H^ and found that only *R175H-#4* significantly reduced p53^R175H^ levels (Supplementary Figure S1B).

### Downregulation of mutp53 in p53^mut^ cancer cells reduces proliferation and migration

**Figure 3:**
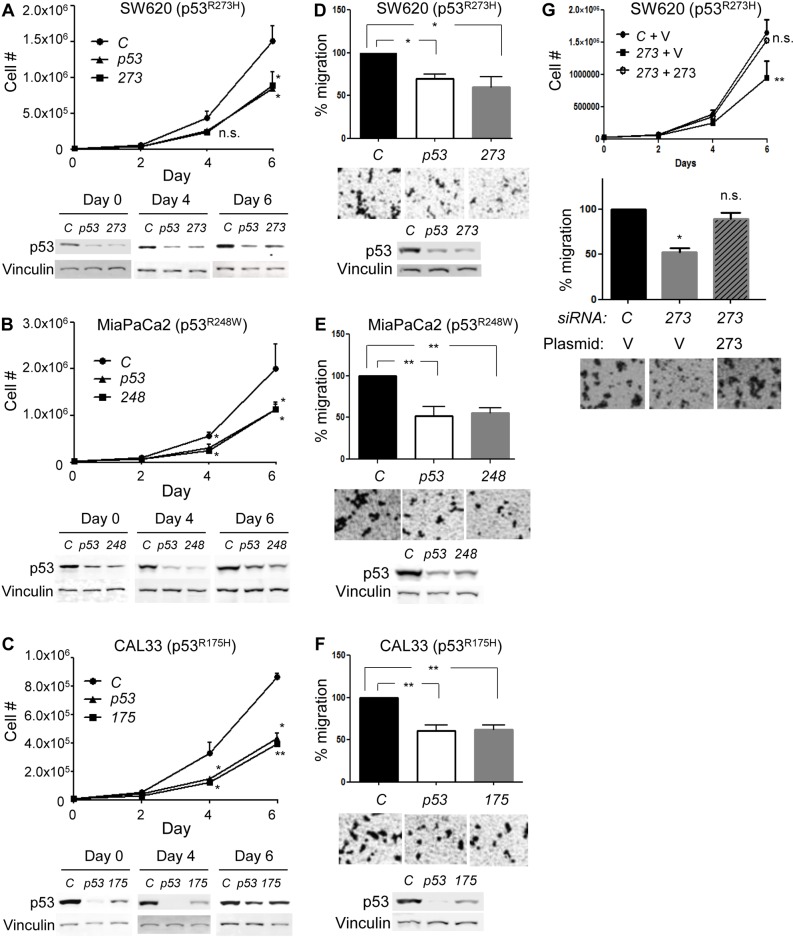
Mutp53 knockdown by mutant-specific siRNAs in cancer cells expressing mutp53 alone (p53^**mut**^) reduces cell proliferation and migration **A.**-**C.** Cell proliferation assays using **A.** SW620 (p53^R273H^), **B.** MiaPaCa2 (p53^R248W^), and **C.** CAL33 (p53^R175H^) cell lines following transfection of indicated siRNAs. Proliferation curves (top) and representative western blotting results for p53 and Vinculin (bottom). **D.**-**F.** Transwell migration assays for 10h using **D.** SW620 (p53^R273H^), **E.** MiaPaCa2 (p53^R248W^), and **F.** CAL33 (p53^R175H^) cell lines following transfection of indicated siRNAs. Graphs showing relative cell migration (%) compared to the number of migrating cells in control. Representative images are placed below the graphs, and representative western blotting results for p53 and Vinculin are below the images **G.** SW620 cells transfected with *control* or *273*-specific siRNAs were infected with control empty (V) or p53^R273H^ (273)-encoding retroviral vectors, followed by cell proliferation (top) and migration (bottom) assays. Data were analyzed as in Figure 3A-F. Error bars: means ± S.D. from three independent experiments. *P< 0.05, **P < 0.01, n.s.: not significant; Student's *t* test.

We next examined whether our developed *mutp53*-specific siRNAs showed any biological effects on human p53^mut^ cancer cells. We first performed cell proliferation assays. When human colorectal adenocarcinoma SW620 cell line endogenously expressing p53^R273H^ was transfected with *non-target#1* (negative control, *C*), *p53* (positive control targeting both wtp53 and mutp53, *p53*) or *R273H-#3* (*273*) siRNAs, proliferation of SW620 cells was reduced by both *p53* and *273* siRNAs, compared with the *control* siRNA (Figure 3A). Downregulation of p53^R273H^ was effective during the course of experiments as shown by western blotting. Similar results were obtained when MiaPaCa2,a pancreatic carcinoma cell line (p53^R248W^, Figure 3B) and CAL33,a tongue squamous cell carcinoma cell line (p53^R175H^, Figure 3C) were transfected with *R248W-#1* (*248*) and *R175H-#4* (*175*) siRNAs, respectively.

We next examined effects of mutp53 downregulation on the migratory potential of the same set of cell lines using the aforementioned siRNAs. In all the three cell lines examined (SW620, MiaPaCa2, and CAL33), *mutp53*-specific siRNAs successfully reduced the migratory potential of cells similar to the *p53* siRNA, as compared with the *control* siRNA (Figure 3D-3F). Thus, mutp53 downregulation resulted in reduced cell proliferation and migration following depletion of GOF p53 mutants, suggesting that oncogenic phenotypes of these cancer cells were, at least partially, dependent on the presence of mutp53. We also confirmed that reduced cell proliferation and migration of SW620 cells by *273* siRNA were substantially rescued by simultaneous overexpression of p53^R273H^ (Figure 3G).

Although we demonstrated biological effects of mutant-specific siRNAs, these siRNAs might have off-target effects. To mitigate this possibility, we first transfected these siRNAs, as well as *control* and *p53* siRNAs, into p53-null HCT116^null/null^ cells and performed cell proliferation and migration assays (Supplementary Figure S2). Results demonstrated that none of the siRNAs altered cellular behaviors of HCT116^null/null^ cells.

Additionally, we performed cell proliferation and migration assays using several cell lines carrying wtp53 (U2OS, HCT116, and SW48, Supplementary Figure S3). As expected, none of the *mutp53*-specific siRNAs altered proliferation or migration of these cell lines, as compared with the *control* siRNA. These results suggest that observed reduction in cell proliferation and migration by *mutp53*-specific siRNAs was not due to off-target effects of these siRNAs.

### Allele-specific silencing of mutp53 in cancer cells expressing both wtp53 and mutp53 reduces proliferation and migration with restoration of wtp53 activity

**Figure 4:**
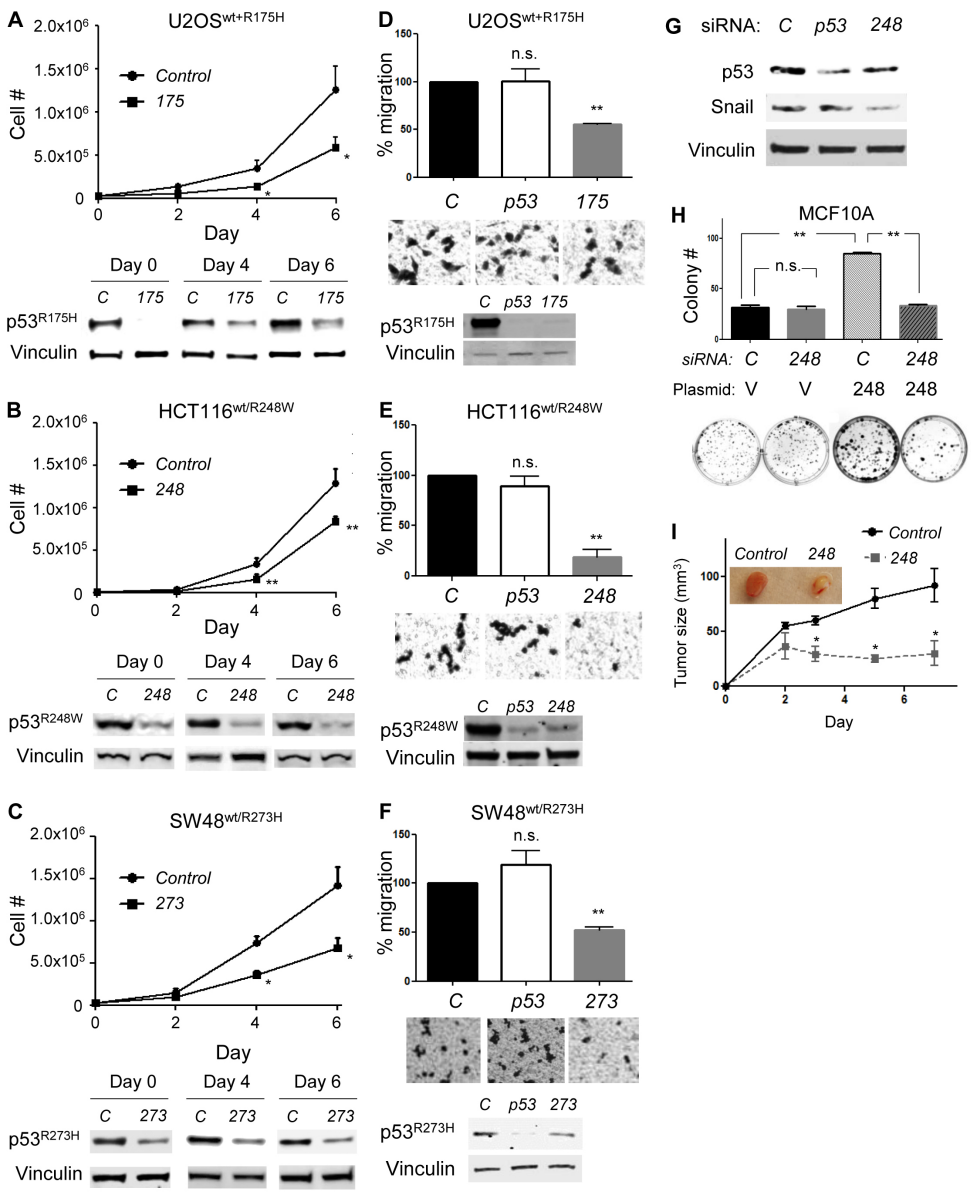
Mutp53 knockdown by *mutp53*-specific siRNAs in cancer cells expressing both wtp53 and mutp53 reduces malignant properties of cancer cells **A.**-**C.** Proliferation assays using **A.** U2OS^wt+R175H^, **B.** HCT116^wt/R248W^, and **C.** SW48^wt/R273H^ cell lines following transfection of indicated siRNAs. Representative western blotting results for p53 and Vinculin are below the graphs of cell proliferation assays. **D.**-**F.** migration assays for 10h using **D.** U2OS^wt+R175H^, **E.** HCT116^wt/R248W^, and **F.** SW48^wt/R273H^ cell lines following transfection of indicated siRNAs. Representative images of migration assays and western blotting are below the graphs of migration assays. **G.** HCT116^wt/R248W^ cells were transfected with *control*, *p53*, or *248* siRNAs, followed by western blotting for p53 and Snail. **H.** MCF10A cells infected with control empty (V) or p53^R248W^ (248)-encoding retroviral vectors were transfected with *control (C)* or *248* siRNAs, followed by colony formation assays for 10 days. Summary results (top) and representative images (below) are shown. **I.** One day after transfection of HCT116^wt/R248W^ cells with *control* or *248* siRNAs, cells (2e6) were subcutaneously injected into nude mice. Tumor sizes were measured at least every 2 days till day 7. Error bars: means ± S.D. from three independent experiments. *P < 0.05, **P < 0.01, n.s.: not significant; Student's *t* test.

Although frequency of *p53* loss of heterozygosity is varied among cancer types, stages of cancer, or the nature of the studies performed [24-26], it is generally appreciated that the *wtp53* allele is retained during early stages of tumor development, but it can be lost or mutated with tumor progression [27-30]. Hence, we wanted to examine the biological effects of *mutp53*-specific knockdown in cancer cells expressing both wtp53 and mutp53. Since oncogenic p53 mutants also have DN effects towards wtp53 [31], we hypothesized that specific depletion of mutp53 could restore the wtp53 activity, leading to efficient suppression of cancer cell progression. To test this hypothesis, we infected U2OS cells with a retroviral vector encoding *p53^R175H^* (U2OS^wt+R175H^) and transfected them with *control* or *175* siRNAs, followed by cell proliferation assays (Figure 4A). We found that p53^R175H^ expression was efficiently inhibited until day 6 (day 7 after transfection) and inhibited cell proliferation was observed at days 4 and 6 (Figure 4A).

We also used genetically engineered colorectal carcinoma cell lines heterozygous for wtp53 and mutp53 (p53^wt/mut^) carrying a point mutation at the endogenous *p53* locus (purchased from Horizon Discovery). The presence of heterozygous mutations at codons 248 and 273 were confirmed in HCT116^wt/R248W^ and SW48^wt/R273H^ cell lines, respectively, by reverse transcription (RT)-PCR and direct sequencing (Supplementary Figure S4). When HCT116^wt/R248W^ and SW48^wt/R273H^ cells were transfected with *248* and *273* siRNAs, respectively, the proliferation of these cells were significantly reduced compared with the *control* siRNA, similar to those observed in U2OS^wt+R175H^ cells (Figure 4B and 4C).

We next performed transwell migration assays using the same set of cell lines as Figure 4A-4C (Figure 4D-4F). In the migration assays, we included *p53* siRNA to downregulate both wtp53 and mutp53 and examined whether altered migration by *mutp53*-specific siRNAs, if detected, was caused either by loss of mutp53 or by the remaining wtp53. Results demonstrated that migration of cancer cells expressing both wtp53 and mutp53 was inhibited only when *mutp53*-specific siRNAs were transfected (Figure 4D-4F). Indeed, p53^R248W^ knockdown in these cells reduced expression of Snail, a migration marker degraded by the p53-MDM2 axis [32], whereas knockdown of both wtp53 and mutp53 by *p53* siRNA did not alter the expression of this protein obviously (Figure 4G).

Additionally, we demonstrated that overexpression of p53^R248W^ increased colony forming potential of an immortalized mammary epithelial cell line MCF10A (p53^wt/wt^), which was significantly reduced by transfection of *248* siRNA (Figure 4H). Moreover, when HCT116^wt/R248W^ cells were subcutaneously injected into nude mice following transfection with *248* siRNA, the tumor forming potential was significantly decreased as compared with those with *control* siRNA (Figure 4I). Together, these results suggest the possibility that knockdown of oncogenic mutp53 in cancer cells expressing both wtp53 and mutp53 restored the wtp53 activity, leading to suppression of their aggressive properties.

### Allele-specific silencing of mutp53 by siRNAs induces apoptosis and/or proliferation arrest with restoration of wtp53 activity

**Figure 5:**
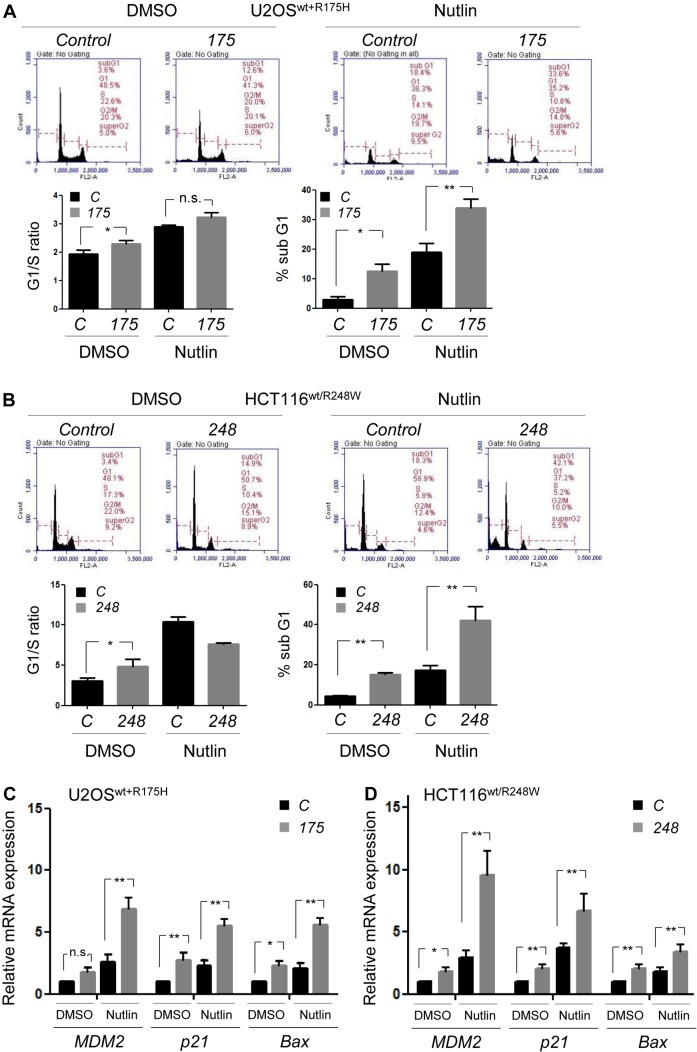
Allele-specific silencing of mutp53 by siRNAs induces apoptosis and proliferation arrest with restoration of wtp53 activity **A.**, **B.** PI staining and flow cytometry using U2OS cells infected with p53^R175H^ (U2OS^wt+R175H^, **A.** or HCT116^wt/R248W^ cells **B.** Two days after transfection with *mutp53*-specific siRNAs, cells were treated with either DMSO or 5 μM of Nutlin-3a (Nutlin) for 48h. Representative results of flow cytometry (above) and summarized graphs (below), showing G1/S ratio and sub-G0/G1 apoptotic fraction. **C.**, **D.** qRT-PCR using U2OS^wt+R175H^ **C.** or HCT116^wt/R248W^ **D.** cells, for p53 target genes (*MDM2, p21*, and *BAX*) and *GAPDH*, following mutp53 knockdown by indicated siRNAs and treatment with either DMSO or 5 μM of Nutlin for 24h. Data are presented as relative values to the DMSO-treated, *control* siRNA-transfected group normalized by the value to *GAPDH*. Error bars: means ± S.D. from three independent experiments. *P < 0.05, **P < 0.01, n.s.: not significant; Student's *t* test.

To understand the mechanisms by which specific downregulation of mutp53 in cancer cells expressing both wtp53 and mutp53 led to reduced tumor progression, PI staining and flow cytometry were performed using U2OS^wt+R175H^ (Figure 5A) and HCT116^wt/R248W^ (Figure 5B) cell lines, in the absence or presence of Nutlin-3a, an inhibitor of the MDM2-p53 interaction. Since we found reduced cell proliferation at day 4, we performed cell cycle analyses 96h after transfection of *mutp53*-specific siRNAs. In both the cell lines, allele-specific silencing of p53 mutants led to significant increase in cellular population of sub-G0/G1 phase of the cell cycle (indicating apoptosis) and modest increase in G1/S ratio (indicating proliferation arrest) (Figure 5A, 5B). Apoptosis became more obvious in both the cell lines, when cells were treated with Nutlin-3a, which was furthermore profound upon mutp53 downregulation (Figure 5A, 5B). These results suggest possible restoration of wtp53 activity following depletion of mutp53 in cells expressing wtp53 and mutp53, as well as cooperative effects of mutp53 knockdown with a p53-activating drug, Nutlin-3a, thus causing induction of cell cycle arrest and apoptosis.

We then performed quantitative RT-PCR (qRT-PCR) for p53 target genes, *MDM2, p21, and BAX*, to examine whether knockdown of mutp53 led to wtp53 activation in these cells with or without treatment with Nutlin-3a for 24h (Figure 5C, 5D). Expression of mRNAs for most of the p53 target genes was significantly upregulated following mutp53 downregulation, which became more profound when cells were treated with Nutlin-3a in both the cell lines, implying cooperative effects of mutp53 knockdown with Nutlin-3a (Figure 5C, 5D). These results strongly suggest that depletion of mutp53 in cells expressing both wtp53 and mutp53 restored wtp53 activity and subsequently induced apoptosis and cell cycle arrest.

### Allele-specific silencing of mutp53 increases doxorubicin sensitivity by restoration of wtp53 activity

**Figure 6:**
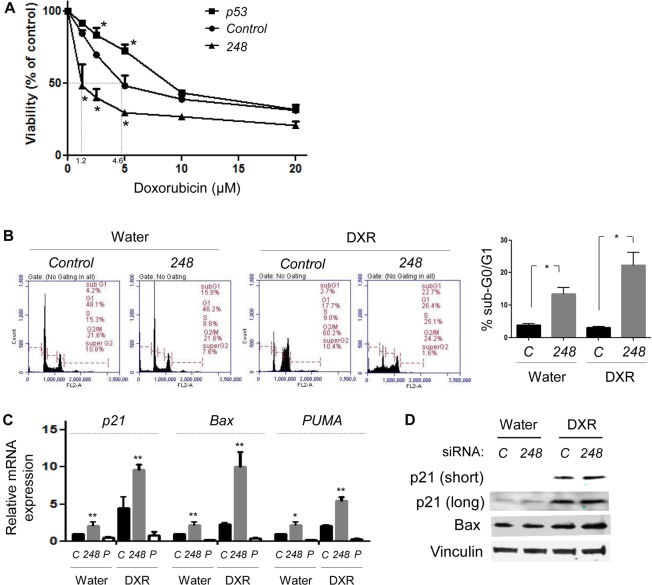
Allele-specific silencing of mutp53 increases doxorubicin sensitivity by restoration of wtp53 activity **A.** MTT assays. HCT116^wt/R248W^ cells transfected with *control*, *p53*, or *248* siRNAs were treated with water (control) or varying concentrations of doxorubicin for 48h, followed by MTT assays. **B.** PI staining and flow cytometry, using cells transfected with *control* or *248* siRNA and treated with water (control) or 1.25 μM of doxorubicin (DXR) for 24h. Representative results of flow cytometry (left) and summarized graphs showing sub-G0/G1 apoptotic fraction (right). **C.** QRT-PCR for p53 target genes, *p21, BAX*, and *PUMA*, using HCT116^wt/R248W^ cells transfected with *control (C)*, *248, or p53 (P)* siRNAs with treatment of water or DXR for 24h. Data are presented as relative values to the water-treated, *control* siRNA-transfected group normalized by the value to *GAPDH*. **D.** Western blotting for p21, BAX, and Vinculin following treatment of *control* (*C*) or *248* siRNA-transfected HCT116^wt/R248W^ cells with water or DXR treatment for 24h. Short: short exposure, Long: long exposure. Error bars: means ± S.D. from three independent experiments. *, *P* < 0.05 and **, *P* < 0.01; Student's *t* test.

The major purpose of developing strategies to deplete mutp53 is to increase the efficacy of current therapeutics. Towards this goal, we examined the effects of *mutp53*-specific knockdown on doxorubicin sensitivity of HCT116^wt/R248W^ cells. Results from MTT assays following treatment with various concentrations of doxorubicin for 48h revealed that allele-specific silencing of mutp53 in HCT116^wt/R248W^ cells significantly increased sensitivity to doxorubicin (IC50 value from 4.6 μM to 1.2 μM, Figure 6A). However, knockdown of both wtp53 and mutp53 in HCT116^wt/R248W^ cells did not sensitize cells, and rather modestly decreased doxorubicin sensitivity. These results suggest that increased drug sensitivity was caused by restoration of wtp53 activity, but not loss of oncogenic mutp53.

We also performed PI staining and flow cytometry to examine the cooperative effects of mutp53 knockdown with doxorubicin (DXR) on cell viability. DXR treatment in HCT116^wt/R248W^ cells at 1.25 μM (near IC50 value of *248* siRNA-transfected cells) caused G2/M arrest of the cell cycle (Figure 6B). However, when HCT116^wt/R248W^ cells were exposed to DXR following specific downregulation of p53^R248W^, significant increase in the apoptotic cell population was observed (Figure 6B). These results suggest that allele-specific silencing of mutp53 by siRNAs increases DXR sensitivity.

To understand the mechanisms by which mutp53 knockdown increased DXR-mediated cell death, we performed qRT-PCR using HCT116^wt/R248W^ cells with or without DXR treatment following transfection of *control (C)*, *248*, and *p53 (P)* siRNAs. Consistent with the results of flow cytometry, specific downregulation of p53^R248W^ in HCT116^wt/R248W^ cells increased mRNA expression of all the p53 target genes (*p21, BAX*, and *PUMA*), as compared with *control* siRNA-transfected cells (Figure 6C). Increased mRNA expression of p53 target genes became much more obvious when p53^R248W^-knockdown cells were treated with DXR (Figure 6C). As expected, knockdown of both wtp53 and mutp53 by *p53* siRNA did not alter mRNA expression of these genes regardless of DXR treatment (Figure 6C). We also confirmed concomitant increase in protein levels of p21 and BAX upon knockdown of p53^R248W^ with or without DXR treatment (Figure 6D). These results strongly suggest that *mutp53*-specific knockdown in p53^wt/mut^ cells restored transcriptional function of wtp53, due to loss of DN activity of mutp53, thereby increasing sensitivity of cancer cells to DXR.

Taken together, allele-specific silencing of mutp53 reduced proliferation and migration of cancer cells expressing both wtp53 and mutp53, and also enhanced their DXR sensitivity along with restoration of wtp53 activity.

## DISCUSSION

Mutp53 is one of the best “druggable” targets, since it is uncommon in normal human tissues but is detected in approximately 50% of human cancers. Given that the majority of hotspot p53 mutants are oncogenic, strategies that deplete only mutp53, but not wtp53, should be developed as targeted cancer therapies.

Increasing evidence suggests that depletion of oncogenes in tumors results in attenuated tumor malignancy and progression, indicating that cancer cells are addicted to oncogenes [33]. Depletion of K-RAS(G12D) and H-RAS(G12V) by their specific shRNA-encoding viral vectors led to reduced tumor growth of pancreatic and ovarian cancers, respectively [34, 35]. Downregulation of EGFR by an *EGFR*-specific shRNA lentiviral vector decreased cell proliferation and invasion of gastric cancer with induction of cell cycle arrest and apoptosis [36]. Furthermore, silencing of Her2/Neu oncogene in breast cancer cells successfully inhibited cell proliferation with induction of apoptosis [37, 38]. Thus, survival and proliferation of many cancers are dependent on the presence of oncogenes [10-14, 39]. During the late stage of tumorigenesis, cancer cells frequently lose the *wtp53* allele and only express mutp53. Even in this context, strategies that deplete mutp53 should still attenuate tumor progression as discussed above. Indeed, our results demonstrate that mutp53 silencing in multiple types of p53^mut^ cancer cells (osteosarcoma, colorectal carcinoma, head & neck squamous cell carcinoma, pancreatic carcinoma) leads to inhibition of cell proliferation and migration.

During the early stage of tumorigenesis, mutations in the *p53* gene mostly occur only in one allele and hence tumors retain the *wtp53* allele [40]. In this context, depletion of mutp53 would be more effective for cancer treatment, because it eliminates the DN activity of mutp53 and restores the wtp53 activity to suppress tumor progression. Our studies using cancer cells expressing both wtp53 and mutp53 provide evidence that mutp53-specific knockdown in these cells leads to reduced cell proliferation and migration due to loss of DN mutp53 and subsequent restoration of wtp53 activity. This is further confirmed by upregulation of several p53 downstream target genes. Furthermore, our results indicate that mutp53-specific knockdown increases sensitivity of p53^wt/mut^ cancer cells to a chemotherapy drug, doxorubicin. To the best of our knowledge, this is the first study to demonstrate biological effects of *mutp53*-specific siRNAs on cancer phenotypes.

Our results also show that treatment of cancer cells expressing both wtp53 and mutp53 with Nutlin-3a or doxorubicin results in some activation of wtp53 with reduced cell proliferation. Similar observations are previously reported [25, 41, 42]. Also, it should be noted that even incomplete knockdown of mutp53 in these cells is sufficient to restore wtp53 activity, despite the fact that mutp53 levels are much higher than wtp53. These observations may suggest that effects of DN activity of mutp53 on wtp53 are not robust and even incomplete knockdown of mutp53 would allow some wtp53 to be released from the mutp53-wtp53 oligomers which might be sufficient to restore wtp53 activity, hence leading to tumor suppression. Indeed, it is reported that DN mutp53 is ineffective in impairing the transcriptional activity of wtp53, where at least three mutants are required to inactivate a tetramer [42]. These observations underscore a complexity of wtp53 and mutp53 function when both alleles are simultaneously present in cells.

Our finding of reduced malignant properties of cancer cells by the allele-specific mutp53 silencing is a stepping stone in the development of targeted cancer therapies. Besides our study of the siRNA mediated-mutp53 inactivation, pharmacological approaches of depletion or reactivation of mutp53 have been reported. Some of these mutp53-targeting small molecules are currently under clinical trials [43]. When compared with siRNA approaches, these small molecules can be conveniently delivered to tumor cells. However, one major drawback of pharmacological approaches is that they often have unexpected biological effects associated with specificity issues [43, 44]. Additionally, small molecules could sometimes result in the selection of cells resistant to them [45, 46]. On the other hand, siRNA-based approaches are specific to the target mRNA and can distinguish sequences with just one base difference, if it does not induce off-target effects [47]. The siRNA approach could be used to overcome treatment of cancer cells that have become resistant to small molecules. However, the major hurdles of the siRNA approach are efficacy associated with siRNA delivery to tumor cells and *in vivo* stability of siRNAs. Methodologies that specifically deliver siRNAs into tumors *in vivo*, such as surface-modified nanoparticle-mediated siRNA delivery, should be further developed to improve the efficacy and specificity of targeted cancer therapies by siRNAs [48-51]. If the specificity for mutp53 is high with minimal effects on wtp53, then they are expected to cause reduced side effects, since they will potentially not affect normal cells having wtp53.

Various *in vivo* and translational studies would be the next step before its clinical use. The pathways altered by downregulation of mutp53 in p53^mut^ cells and the underlying mechanisms also remain to be elucidated. Apart from the three designed siRNAs for hotspot p53 mutations, there are many other mutations that need to be tested. Each mutation requires designing of its own specific siRNA. It would also be important to test whether pools of various *mutp53*-specific siRNAs could only deplete mutp53 with little effect on wtp53. This might enable us to treat various tumors carrying different p53 mutations with the pooled *mutp53*-specific siRNAs.

## MATERIALS AND METHODS

### Cell lines

All of the following human cell lines (with different p53 status) were maintained in Dulbecco's Modified Eagle's Medium (DMEM) or Roswell Park Memorial Institute (RPMI) medium with 10% fetal bovine serum (FBS) and 1% penicillin-streptomycin: human osteosarcoma KHOS/NP (p53^R156P^), U2OS (p53^wt^), colorectal carcinoma HCT116 (p53^wt^), colorectal adenocarcinoma SW48 (p53^wt^), tongue squamous cell carcinoma CAL33 (p53^R175H^), pancreatic carcinoma MiaPaCa2 (p53^R248W^), colorectal adenocarcinoma SW620 (p53^R273H^), and mouse osteosarcoma 318-1 (p53^R172H^) as described in [21]. MCF 10A (p53^wt^), a normal mammary epithelial cell line, was grown in DMEM/F12 supplemented with 10% fetal bovine serum (FBS), 1% penicillin-streptomycin, EGF (20 ng/ml), hydrocortisone (0.5 mg/ml) and cholera toxin (100 ng/ml). HCT116^wt/R248W^ and SW48^wt/R273H^ cell lines, which were genetically engineered to have a heterozygous mutation at the endogenous *p53* locus, were purchased from Horizon Discovery Group plc (Cambridge, United Kingdom). When required, cells were treated with 5 μM of a MDM2 inhibitor Nutlin-3a (Sigma-Aldrich, St. Louis, MO) or doxorubicin at different concentrations (Cayman Chemical, Ann Arbor, Michigan) for 24 to 48h to activate p53 [52, 53].

### siRNA transfection

Transfection of siRNAs (40-80 nM) was performed with INTERFERin^®^ according to the manufacturer's protocol (Polyplus-transfection Inc., New York, NY). Double strand siRNAs were purchased from Integrated DNA Technology (Coralville, Iowa). In all experiments, *non-target#1* siRNA (GE Healthcare Life Sciences, Lafayette, CO) was used as a negative control. As a positive control to downregulate both wtp53 and mutp53, the following siRNA target sequence was used: *GAGAUGUUCCGAGAGAGCUGAUU*.

### Sphere and tumor formation assays

Cancer cells were infected with control vectors or *p53* shRNA-encoding lentiviral vectors (shp53 pLKO.1 puro for human p53 and pSicoR p53 for mouse p53, Addgene, Cambridge, MA, USA). Sphere formation assays were performed as previously described [54]. Briefly, cells (20 cells per well) were plated on 96-well ultra-low attachment plates (Corning Inc., Corning, NY, USA) under serum-free sphere-specific conditions for 10-14 days and numbers of spheres were counted. Sphere forming potential was calculated as # of spheres formed/# of cells seeded.

KHOS/NP cells infected with *control* or *p53* shRNA lentiviral vectors were subcutaneously injected into NIH-III nude mice (Charles River Laboratories, Wilmington, MA). Tumor sizes were measured three-dimensionally 3 to 4 times a week for 3 weeks. Similarly, HCT116^wt/R248W^ cells transfected with *control* or *248*-specific siRNAs were subcutaneously injected into NIH-III nude mice and tumor sizes were measured three-dimensionally every 2 days for a week.

### Western blotting

Cells were lysed with radioimmunoprecipitation assay (RIPA) buffer containing phosphatase and protease inhibitors (EMD Chemicals, San Diego, CA). Cell lysate containing 20-100 μg of protein was loaded onto 4-12 % tris-glycine gel (Bio-Rad Laboratories, Inc, Hercules, CA), separated by electrophoresis, transferred to polyvinylidene fluoride (PVDF) membrane (GE Healthcare Life Sciences), blotted with primary antibodies against specific proteins, and appropriate secondary antibodies conjugated with fluorescence. All blots were analyzed with the Li-Cor Odyssey infra-red imaging systems (Lincoln, Nebraska). The following antibodies were used: p53: DO1, Snail: H-130, p21: F-5, Bax: N-20, GAPDH: H-12 (Santa Cruz Biotechnology, Dallas, Texas, USA), Vinculin: 10R-C105a (Fitzgerald, Acton, MA), and IRDye 800CW goat anti-mouse IgG (LI-COR).

### Proliferation assays

Twenty four (24) h after transfection, cells were seeded onto 6-well plates (10,000-30,000 cells per well depending on the cell line, day 0). Live cell numbers were counted at days 2, 4 and 6 following trypan-blue staining.

### Migration assay

Migration assays were performed using 24-well transwell chambers (6.5 mm diameter, 8 μm pore size; Corning Inc., Corning, NY). Forty two (42) h after transfection of siRNAs, cells were suspended in 0.5% FBS-containing media. Cells (5,000-25,000 depending on the cell line) in 100 μl of 0.5% FBS-containing DMEM was added into the upper compartment of the chamber, while 10% fetal bovine serum in DMEM was added to the lower compartment as chemoattractant. Cells were then allowed to migrate across the membrane by incubating at 37°C in CO_2_ incubator for 10h [55]. The non-migrating cells were removed from the upper face of the filters using cotton swabs, while migrating cells to the lower face of the filters were fixed and stained with Diff-Quik Stain Set (Dade Behring, Newark, DE). Stained cells in the entire fields were counted.

### MTT assay

Twenty four (24) h after transfection, cells (10,000 cells) were seeded onto a 96-well plate. Twenty four (24) h later, cells were treated with varying concentrations of doxorubicin (0, 1.25, 2.5, 5, 10, and 20 μM) for 48h, followed by standard MTT assays [56]. Briefly, after cells were incubated with 5 mg/ml of MTT for 3h, the media was replaced with DMSO for 15 min of incubation with shaking in the dark. Results were obtained by reading the plate at 570 nm [56].

### Propidium iodide (PI) staining and flow cytometry

To perform cell cycle analysis, cells were fixed with 70% ethanol at −20°C and stained with PI solution (Life Technologies) in the presence of 62 μg/ml RNase A, followed by flow cytometric analysis using BD Accuri flow cytometer (BD biosciences, San Jose, CA).

### Quantitative reverse transcription PCR (qRT-PCR)

RNA was isolated using the RNA-Quick MiniPrep (Zymo Research, Irvine, CA). Total RNA (1 μg) was reversed transcribed to cDNA using M-MLV reverse transcriptase (Amresco, Solon, OH), according to the manufacturer's instructions, and TaqMan assays were performed with ViiA7 (Life Technologies, Foster City, CA). TaqMan assay primers and probes were purchased from Life Technologies using the following assay numbers: MDM2, Hs00242813_m1; p21, Hs00355782_m1; BAX, Hs00180269_m1; PUMA, Hs00248075_m1. The mRNA levels were normalized to those of GAPDH.

### Immunohistochemistry (IHC)

The tumor tissues derived from subcutaneously injected KHOS/NP were harvested and subjected to IHC for Ki-67: H-300 (Santa Cruz Biotechnology) and cleaved caspase-3: D3E9 (Cell Signaling).

### Colony formation assay

Cells were plated on 6-well plates (500 cells per well) 24h after transfection with *control* or *248*-siRNAs. Colonies were allowed to grow for 10 days, then fixed with methanol, and stained with 0.1% of crystal violet.

### Statistical analysis

The differences in cell proliferation, migration, survival, and gene expression between different samples and/or treatments were analyzed by two-tailed Student's *t*-tests with GraphPad Prism 5 (GraphPad Software, Jolla, CA). Statistical significance was set at *p* < 0.05, unless otherwise stated in the text.

## SUPPLEMENTARY MATERIAL AND FIGURES


