# Supplementary material for: Allele-specific silencing of mutant p53 attenuates dominant-negative and gain-of-function activities
Source: Oncotarget. 2015 Dec 16;7(5):5401–15. doi: 10.18632/oncotarget.6634 (PMC4868694; doi:10.18632/oncotarget.6634)
Supplement: Supplementary file 1 [file oncotarget-07-5401-s001.pdf]

Allele-specific silencing of mutant p53 attenuates dominant-negative and gain-of-function activities

Supplementary Material

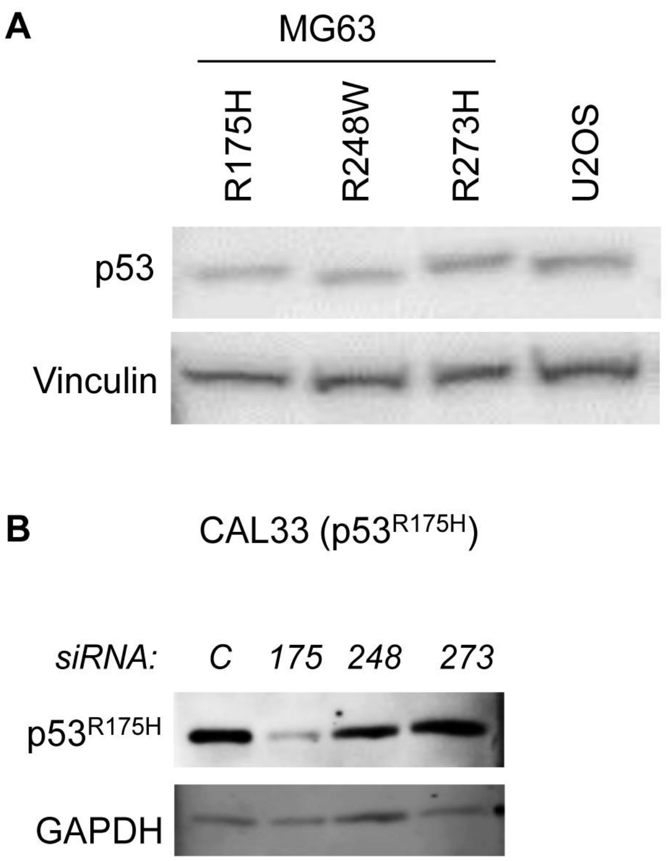

Supplementary figure S1. (A) Western blotting for p53 and vinculin using MG63-subcell lines exogenously expressing hotspot p53 mutants ($p53^{R175H}$, $p53^{R248W}$, $p53^{R273H)}$, as well as U2OS cells endogenously expressing wild-type p53. (B) Western blotting for p53 and GAPDH, following transfection of CAL33 with *Control*, R*175H-#4 (175)*, R*248W-#1 (248)*, and R*273H-#3 (273)* siRNAs.

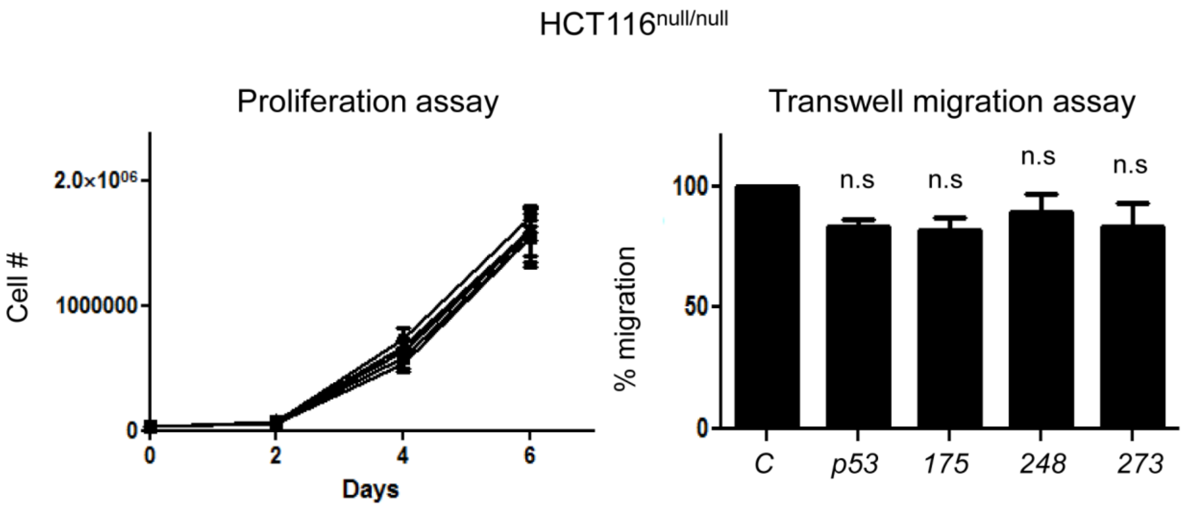

Supplementary figure S2. Cell proliferation (left) and migration (right) assays following siRNA transfection of *Control (C)*, *p53*, *R175-#4 (175)*, *R248-#1 (248)*, and *R273-#3 (273)* siRNAs in HCT116$^{null/null}$ cells. n.s.: not significant; Student's *t*-test.

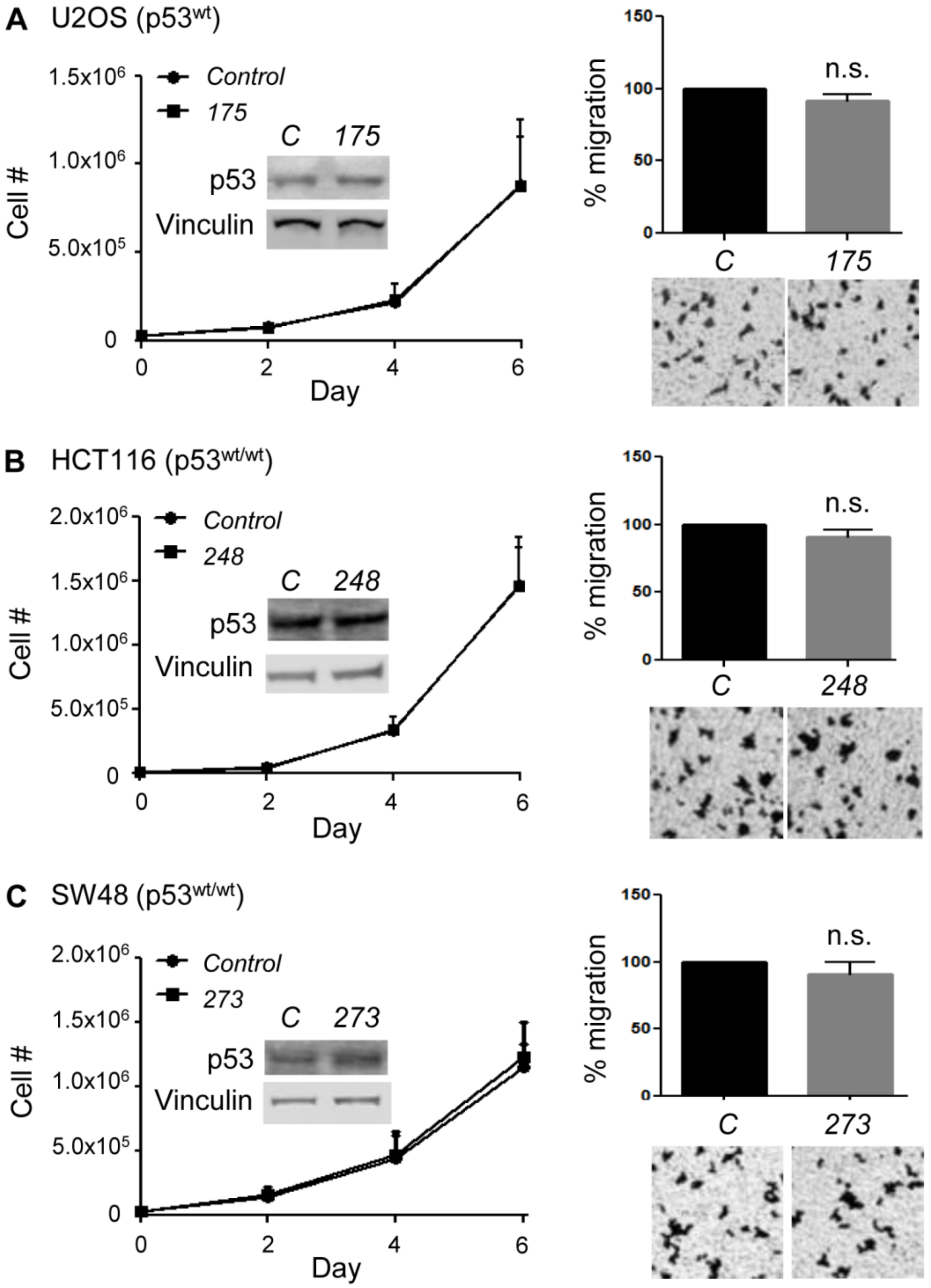

Supplementary Figure S3. Proliferation (left) and migration (right) assays using cell lines wild-type for p53 following transfection of control or mutant-specific siRNAs. **A**) U2OS, **B**) HCT116, and **C**) SW48 cell lines. SiRNAs for $p53^{R175H}$, $p53^{R248W}$, and $p53^{R273H}$ did not alter proliferation and migration. Representative western blotting for p53 and Vinculin are present alongside the graphs of cell proliferation assays (left). Summary of migration assays (right) and representative pictures of migration assays below the graphs. Error bars: means ± S.D. from three independent experiments. n.s.: not significant.; Student's t test.

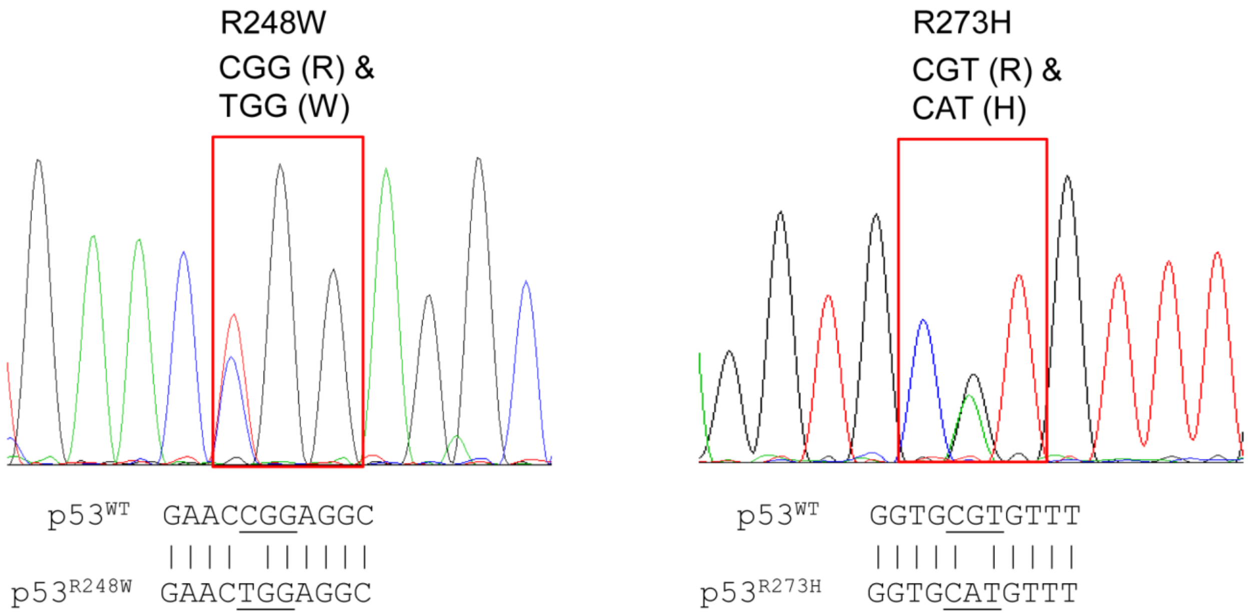

Supplementary Figure S4. Sequencing results for p53 in p53$^{W/M}$ cells using PCR and sequencing primers as follows: PCR-F, ATGGAGGAGCCGCAGTCAGAT; PCR-R, TCAGTCTGAGTCAGGCC; Seq-F, CCATCTACAAGCAGTCACAGCACATG; Seq-R, GCACCACCACACTATGTCGAAAAGTG. The entire *p53* cDNA was amplified by RT-PCR using mRNA from HCT116$^{wt/R248W}$ and SW48$^{wt/R273H}$ cells, followed by sequencing. Results demonstrate one point mutation in each cell line.
